# Impacts of seed priming with salicylic acid and sodium hydrosulfide on possible metabolic pathway of two amino acids in maize plant under lead stress

**DOI:** 10.22099/mbrc.2018.29089.1317

**Published:** 2018-06

**Authors:** Roya Zanganeh, Rashid Jamei, Fatemeh Rahmani

**Affiliations:** Department of Biology, Faculty of Science, Urmia University, Urmia, Iran

**Keywords:** Amino acids metabolism, Hydrogen sulfide, Pb stress, Salicylic acid, *Zea mays* L

## Abstract

Heavy metals pollution is one of the key environmental problems. In this research, the effect of seed priming with salicylic acid and sodium hydrosulfide was investigated on methionine and arginine amino acids contents and some compounds derived from their metabolism as well as *ZmACS6* and *ZmSAMD* transcripts levels in maize plants under lead stress. For this purpose, maize seeds were soaked in salicylic acid (0.5 mM) and sodium hydrosulfide (0.5 mM) for 12 hours and then exposed to lead (2.5 mM) for 9 days. The results showed that lead stress reduced nitric oxide content and shoot *ZmACS6* and *ZmSAMD* transcript levels while increased glycine betaine, methionine, arginine and proline amino acids contents as well as root *ZmACS6* and *ZmSAMD* transcript levels. Salicylic acid and sodium hydrosulfide pretreatments reduced methionine, arginine and proline accumulation and increased glycine betaine and nitric oxide contents and regulated the expression of *ZmACS6* and *ZmSAMD *genes (genes participating in methionine metabolism) under lead stress. Our data suggest that salicylic acid and hydrogen sulfide play role in regulating the methionine and arginine metabolism in maize under lead stress condition.

## INTRODUCTION

Lead (Pb) with atomic number of 82 and atomic mass of 207.2 is one of the most important heavy metals.Since a long time ago, it was considered as one of the environmental pollutants. Growth and metabolism of plants are affected by the increase of this metal in the environment [1]. Pb stress has different effects on the physiological stages of plant. For example, this stress increases the reactive oxygen species (ROS) production, changes plant growth, nitrogen metabolism, and phytohormones biosynthesis, and reduces the efficiency of photosynthesis [2, 3]. One of the consequences of heavy metal stress in plants is the alteration in the nitrogen metabolism and cellular pools of amino acids. The involvement of amino acids in response to stress was demonstrated in several studies.In addition, amino acids play key roles in plants as precursor for the synthesis of various protective compounds [4, 5]. 

Salicylic acid (SA) and hydrogen sulfide (H_2_S) are two signaling molecules with multiple functions in plant growth, development and stress tolerance [6, 7]. The relationship between these two signaling molecules [8] and influence of SA on amino acids contents has been reported in several plants under abiotic stress [9, 10].

In literature, there is limited number of papers regarding the effect of NaHS application on free amino acids under stress condition [11]. In the past, the amelioration of heavy metal stresses including Pb stress have been reported by exogenous SA and H_2_S [12, 3]. The aim of this study was to investigate the effect of SA and NaHS seed priming on the content of certain compounds derived from metabolism of methionine and arginine including glycine betaine (GB), nitric oxide (NO) and proline and transcript level of 1-aminocyclopropane-1-carboxylate synthase (*ZmACS6*) and S-adenosylmethionine synthase (*ZmSAMD*) genes, involved in metabolism of methionine.

## MATERIALS AND METHODS

Seeds of (*Zea mays* L. cv. 704) were supplied by the Agriculture Research Center of Kerman. Seeds were surface sterilized in 10% (w/v) sodium hypochlorite, rinsed several times in distilled water and pretreated with 0.5 mM SA and NaHS for 12h (concentration was optimized in preliminary experiment). After 12 h, seeds were germinated on moistened filter paper for 3 days. Thereafter, seedlings were sown individually in each pot containing sand and perlite in the ratio of 2:1. Plants were grown under long day conditions with 16 h/8 h day/night cycle at a temperature between 20-29°C. The humidity of growth chamber was set to 60-80%. The 6-day old seedlings were subjected to lead stress by 2.5 mM Pb(NO_3_)_2_. After 9 days’ treatment, plants were harvested and immediately stored at -80°C for future analysis. 

**Measurements of GB, NO and amino acids content: **GB content was measured adopting the method of Grieve and Grattan [13] by estimating betaine eperiodite complex. The Zhou et al. [14] method was followed for the estimation of NO content. Amino acids content was determined by the method of Stines et al., [15]. 

**RNA isolation, cDNA synthesis and RT-PCR conditions: **Total RNA was extracted from leaf and root tissues using Louime et al. [16] method with minor modification. cDNA was synthesized from total RNA using a cDNA Kit (Fermentas) according to the manufacturer’s instructions. The cycling protocol for 20 µL reaction mix was 5 min at 65°C, followed by 60 min at 42°C, and 5 min at 70°C to terminate the reaction. Forward and reverse primers sequences used for RT-PCR are given in Table 1. PCR condition was as following protocol: initial denaturation at 95°C for 3 min, followed by 35 cycles at 95°C for 45 s, 58-64°C for 30 s and 72°C for 30 s and final extension at 72°C for 10 min. The products of RT-PCR were separated on 1.5% agarose gel containing Ethidium Bromide (0.5 µg/ml) and visualized and measured using Gel Doc system (Ingenius3, Syngene, UK). The primers were designed using Primer Express 2.0 software (Applied Biosystems).

**Table 1:** Primers used for RT-PCR study

Genes		References
*Actin*	F: 5'-ATTCAGGTGATGGTGTGAGC-3' R: 5'-CTGTACTTCCTTCTAGGTGG-3'	[19]
*ZmACS6*	F: 5'-TCATCACCAACCCTTCCAAC-3' R: 5'-AGTATATCTCGTCGCTCACCA-3'	This study
*ZmSAMD*	F: 5'-CAAGCTCCTGCTCACCATTC-3' R: 5'-TGCAGCAACTTCCTCAGAGA-3'	This study

**Statistical analysis: **All the assays were carried out in triplicate. The results are expressed as mean values and standard error (SE) of the mean. Data analyses were performed using SPSS software version 19 and the means were compared using Duncan’s multiple range (DMRT) test at p < 0.05 following analysis of variance (ANOVA).

## RESULTS

Increase in GB content was detected in shoot (46%) and root (52%) of Pb treated maize plants compared with control, while NO content decreased in both shoot and root tissues by 25 and 20%, respectively. Compared to Pb stress alone, pretreatments with SA and NaHS resulted in increase in GB and NO contents. In non-stressed plants, NaHS pretreatment significantly enhanced shoot GB content while pretreatment of seeds with SA had no significant effect on GB content under normal condition. Both SA and NaHS pretreatments had no significant effect on NO content under normal condition (Table 2).

**Table 2:** Effects of SA and NaHS on GB and NO content in shoots and roots of maize under Pb stress.

Parameters	**Tissue**	**Control**	SA (0.5 mM)	NaHS (0.5 mM)	Pb (2.5 mM)	Pb+SA	Pb+NaHS
GB content (µg/g FW)	Shoot Root	3.61±0.48^d^ 4.57±0.80^c^	4.94±0.52^cd^ 5.18±0.52^c^	5.93±0.61^bc^ 4.87±0.80^c^	6.70±0.23^ab^ 9.66±0.52^b^	7.20±0.30^ab^ 12.66±0.45^a^	7.57±0.47^a^ 10.81±0.82^ab^
NO content (nmol/g DW)	Shoot Root	56.94±3.20^c^ 63.61±2.42^b^	63.42±3.33^bc^ 68.98±2.44^b^	64.53±2.27^bc^ 70.83±3.20^b^	42.68±4.58^d^ 50.46±3.33^c^	73.61±3.20^ab^ 92.31±1.64^a^	76.64±1.95^a^ 86.57±2.45^a^

The result showed that Pb stress caused a marked increase in methionine, arginine and proline contents in both shoot and root tissues. SA decreased methionine content by 89% in shoot and arginine content by 32% in root. SA pretreatment had no obvious effect on arginine content in shoot and methionine content in root. NaHS pretreatment decreased methionine (74%) and arginine (66%) in shoots. However, NaHS increased arginine content by 45% in root and had no significant effect on methionine content in root of Pb stressed plants. Both SA and NaHS pretreatments decreased proline content in both shoot and root parts. Under normal condition, SA pretreatment increased arginine content in both shoots and roots and NaHS pretreatment increased methionine content in root whereas decreased arginine content in shoot.

**Table 3:** Effects of SA and NaHS on methionine, arginine and proline amino acids content in shoots and roots of maize under Pb stress.

**Amino acids** **(µmol/g FW)**	**Tissue**	**Control**	SA (0.5 mM)	NaHS (0.5 mM)	Pb (2.5 mM)	Pb+SA	Pb+NaHS
Methionine	Shoot Root	7.19±0.33^d^ 4.06±0.54^c^	7.11±0.27^d^ 4.79±0.45^c^	7.78±0.32^cd^ 9.64±0.57^a^	108.48±2.59^a^ 6.82±0.51^b^	10.88±0.30^c^ 7.39±0.45^b^	28.09±0.66^b^ 6.86±0.49^b^
Arginine	Shoot Root	154.27±2.89^c^ 24.10±1.15^d^	187.02±4.04^b^ 40.31±1.76^c^	112.83±4.38^e^ 24.47±1.44^d^	413.21±5.77^a^ 52.30±1.75^b^	406.30±3.75^a^ 35.38±2.36^c^	139.07±4.61^d^ 95.12±2.88^a^
Proline	Shoot Root	194.22±2.89^b^ 75.40±2.45^e^	186.09±3.02^b^ 105.40±2.40^c^	110.37±4.08^d^ 211.56±2.52^a^	443.21±4.04^a^ 133.29±2.45^b^	168.69±4.24^c^ 89.35±2.60^d^	186.49±3.32^b^ 86.54±2.59^d^

**Figure 1:**
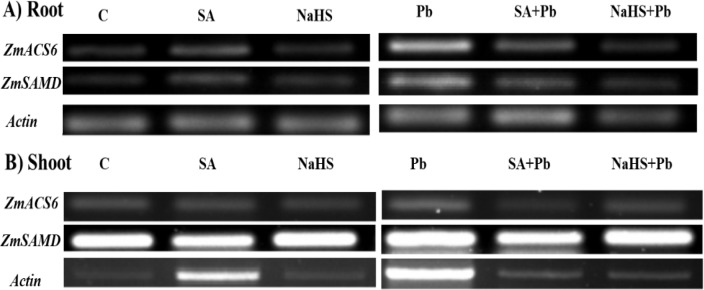
Effects of SA and NaHS pretreatments on expression profile of genes related to proteins in roots (A) and shoots (B) of maize plants treated by 2.5 mM Pb(NO_3_)_2_

**Figure 2:**
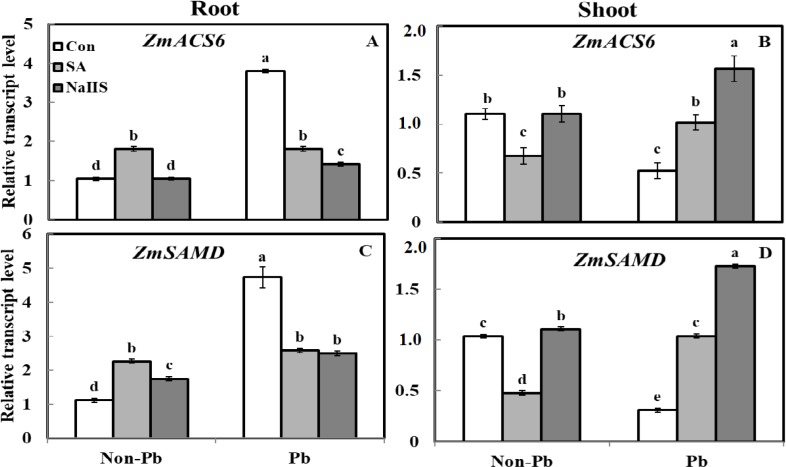
Effect of SA and NaHS on expression of *ZmACS6* and *ZmSAMD* genes in roots and shoots of maize under Pb stress. Different letters indicate significant differences at *P*< 0.05

Under Pb stress, the expression of *ZmACS6 *and* ZmSAMD* genes were strongly induced by 72% (Fig. 1 and 2A) and 76% (Fig. 1 and 2C) in roots while downregulated by 52% (Fig. 1 and 2B) and 70% (Fig. 1 and 2D) in shoots, respectively. When plants were pretreated with SA and NaHS under Pb stress, transcript levels of *ZmACS6* and *ZmSAMD* were decreased in the roots whereas upregulated in shoots of maize plants. However, SA pretreatment of non-stressed plants increased both *ZmACS6* and* ZmSAMD* transcript levels and NaHS pretreatment increased *ZmSAMD* gene expression, significantly. The SA pretreatment declined and NaHS upregulated *ZmACS6* and *ZmSAMD* transcription levels in shoots under normal condition.

## DISCUSSION

The methionine content increased under Pb stress in both shoot and root tissues (Table 3). The same results have been reported by Sujatha and Priyadarshini [5] in pigeon pea plants grown under Pb and cadmium stresses. This induction can be related to the role of this amino acid as the precursor of the ethylene synthesis [6] due to elevation in the transcript level of *ZmACS*6 under Pb stress in roots (Fig. 1). The seed pre-treatment with SA and NaHS alleviated the adverse effects of Pb stress, probably via down-regulation of the *ZmACS6* expression (Fig. 1) in roots and increase in the amount of GB. Decrease of methionine content and upregulation of *ZmSAMD* in shoot also indicates that methionine is diverted toward polyamine biosynthesis in order to detoxification of Pb in SA and NaHS pretreated plants.

Arginine is one of the most important regulators for the developmental, physiological and growth processes of plants [17]. Increasing arginine content can be due to demand for the synthesis of compounds derived from its metabolism as a defense response against stress. However, seed pretreatment with SA and NaHS reduced arginine content under Pb stress conditions. This decline may be due to the positive effect of these compounds on the catabolism of this amino acid for biosynthesis of products derived from its metabolism, which play an important role in improving the stress. Nitric oxide synthase, arginase and arginine decarboxylase are key enzymes in arginine metabolism catalyzing the production of nitric oxide, proline and polyamines, respectively [17]. 

Similar to our obtained results, decline in proline content due to seed pretreatment with SA and NaHS has been reported in rice under copper and cadmium stresses [18, 7] indicating reduced Pb-induced damages. Regarding the reduction of *ZmSAMD* gene expression (polyamine biosynthetic enzyme by methionine) in roots of SA and NaHS pretreated plants, the production of polyamines is probably via arginine decarboxylase enzyme and metabolism of this amino acid. Further investigations are needed to substantiate this suggestion. Since NO content increased in SA and NaHS pretreated plants, arginine metabolism is probably diverted toward NO biosynthesis to improve Pb stress tolerance. 

In this study, Pb stress increased methionine, arginine and proline content as a defense response in maize. SA and NaHS pretreatments improved adverse effects of Pb stress through increase of GB and NO contents and regulation of genes participating in methionine metabolism.
